# Identification of abnormally expressed lncRNAs induced by PM2.5 in human bronchial epithelial cells

**DOI:** 10.1042/BSR20171577

**Published:** 2018-09-12

**Authors:** Xing Li, Mengning Zheng, Jinding Pu, Yumin Zhou, Wei Hong, Xin Fu, Yan Peng, Wenqu Zhou, Hui Pan, Bing Li, Pixin Ran

**Affiliations:** 1 GMU-GIBH Joint School of Life Sciences, Guangzhou Medical University, Guangzhou, Guangdong, China; 2 Gansu Province Hospital of Traditional Chinese Medicine, Lanzhou, Gansu, China; 3 State Key Laboratory of Respiratory Disease, The First Affiliated Hospital of Guangzhou Medical University, Guangzhou, Guangdong, China

**Keywords:** air-liquid-interface, COPD, human bronchial epithelial cells, lncRNAs, PM2.5

## Abstract

To investigate the effect of stimulation of human bronchial epithelial cells (HBECs) by arterial traffic ambient PM2.5 (TAPM2.5) and wood smoke PM2.5 (WSPM2.5) on the expression of long non-coding RNAs (lncRNAs) in order to find new therapeutic targets for treatment of chronic obstructive pulmonary disease (COPD). HBECs were exposed to TAPM2.5 and WSPM2.5 at a series of concentrations. The microarray analysis was used to detect the lncRNA and mRNA expression profiles. Kyoto Encyclopedia of Genes and Genomes (KEGG) pathway analysis and gene ontology (GO) enrichment were conducted to analyze the differentially expressed lncRNAs and mRNAs. Quantitative real-time PCR (qRT-PCR) was performed to confirm the differential expression of lncRNAs. Western blot was performed to study the expression of autophagy and apoptosis-associated proteins. Flow cytometry was used to detect the apoptotic cells. The results indicated that fine particulate matter (PM2.5)-induced cell damage of HBECs occurred in a dose-dependent manner. The microarray analysis indicated that treatment with TAPM2.5 and WSPM2.5 led to the alteration of lncRNA and mRNA expression profiles. LncRNA maternally expressed gene 3 (*MEG3*) was significantly up-regulated in HBECs after PM2.5 treatment. The results of Western blot showed that PM2.5 induced cell apoptosis and autophagy by up-regulating apoptosis-associated gene, caspase-3, and down-regulating autophagy-associated markers, Bcl-2 and LC3 expression. In addition, we demonstrated that TAPM2.5 and WSPM2.5 accelerated apoptosis of human bronchial (HBE) cells, silencing of MEG3 suppressed apoptosis and autophagy of HBE cells. These findings suggested that the lncRNA MEG3 mediates PM2.5-induced cell apoptosis and autophagy, and probably through regulating the expression of p53.

## Introduction

Chronic obstructive pulmonary disease (COPD) is the fourth leading cause of death and constitutes 6% of all global deaths [1]. The World Health Organization (WHO) announced that in 2012 over three million people died of COPD. However, the associated pathogenic mechanism remains unclear. Cigarette smoking and the air pollution are the major risk factors in the development of COPD [2,3]. Fine particulate matter (PM2.5) with an aerodynamic equivalent diameter of less than or equal to 2.5 µm are the atmospheric particles and important component of air pollution. It constitutes heavy metal particles, acidic oxides, organic pollutants, bacteria, fungi, and viruses [4–6]. Studies have shown that it deposits in the airway and lung tissue and trigger abnormal immune inflammatory responses [7–9]. Long-term exposure to traffic and PM2.5 at relatively low levels was associated with lower FEV1 and FVC and an accelerated rate of decline in lung function [10]. It was observed that acute exposure to elevated PM2.5 concentration is associated with acute exacerbation, hospitalization, and mortality due to COPD [11,12].

There is increasing evidence that PM2.5 can induce a series of pathological responses such as oxidative damage, immune and inflammatory responses, cytotoxicity, DNA damages, and gene mutations [13–15]. Recently, studies have suggested that PM2.5 can also affect gene expression [16–18]. However, the associated pathogenic mechanism of PM2.5-induced epigenetic changes and the cell functions remain unclear.

Long non-coding RNAs (lncRNAs) are a class of RNA molecules with length ranging from 200 nt to 100 kb [19]. Previous studies have demonstrated that lncRNAs participate in a range of biological processes such as cell differentiation [20–22]. Expression of many lncRNAs is altered, which is likely to associate with multiple diseases such as neurodegeneration diseases, cardiovascular diseases, cancers, and COPD [23–28]. Recently, lncRNA SAL was found to regulate AECII senescence in the pathogenesis of COPD. However, most of the functions of these lncRNAs and the involvement in the development of COPD remain unclear [29].

In the present study, we treated human bronchial (HBE) cells with arterial traffic ambient PM2.5 (TAPM2.5) and wood smoke PM2.5 (WSPM2.5) and analyzed the expression profiles of lncRNAs. We found that exposure of TAPM2.5 and WSPM2.5 induced the alteration of lncRNAs expression profiles, and caused cell apoptosis and autophagy. In addition, PM2.5-induced cell damage of human bronchial epithelial cells (HBECs) occurring in a dose-dependent manner. Then, Kyoto Encyclopedia of Genes and Genomes (KEGG) pathway analysis and gene ontology (GO) enrichment were conducted for analysis of the differentially expressed lncRNAs and mRNAs. Five up-regulated lncRNAs were selected and identified using quantitative real-time PCR (qRT-PCR). We identified an lncRNA, maternally expressed gene 3 (*MEG3*), whose up-regulated expression mediated cell apoptosis and autophagy by increasing the expression of p53 in HBE cells treated with TAPM2.5 and WSPM2.5. In addition, we proved that the roles of MEG3 on the apoptosis and autophagy of HBE cells and silenced MEG3 inhibited apoptosis and autophagy of HBE cells.

## Materials and methods

### PM2.5 collection

Particle collection and preparation is as described previously [30]. Briefly, PM2.5 was collected from an arterial traffic road and a typical southern kitchen, and then the collections were extracted by DMSO. TAPM2.5 was collected from an arterial traffic road with 13-h sampling time (from 8:00 a.m. local time each day to 9:00 p.m.), from March to April in 2015. The sampling site was set 3 m from the roadside, 2 m above ground. WSPM2.5 samples were collected for 1 h from a typical southern countryside kitchen when the fire was fully burning. The sampling site was set 2 m away from the stove. Subsequently, both TAPM2.5 and WSPM2.5 were collected and extracted.

### Cell culture and exposure to PM2.5

Air–liquid interface (ALI) is an improved respiratory physiology model, which is used widely in *in vitro* to study recapitulate airway epithelial biology morphologically and transcriptionally [31]. Normal human bronchial epithelial (NHBE) cells were purchased from ATCC (VA, U.S.A.) and grown at the ALI as described previously [32]. The second passage of HBEC cells were cultured on collagen gel-coated Transwell chamber (Transwell-Clear, 24-mm diameter, 0.4-µM pore size; Corning3450). Cells with the number 1 × 10^5^ were seeded in the upper compartment incubated in 1 ml Airway Epithelial Cell Basal Medium (BEGM, ATCC), and 2 ml medium was filled in the lower compartment. Cells were used after 21 days in culture, when complete fusion was achieved and the transepithelial resistance was no less than 1000 Ω.cm^2^. Subsequently, TAPM2.5 at a concentration of 57μg/mL, 17.1μg/mL, or 5.7μg/mL was added to HBECs for 6 h. Blank-DMSO (1/250) was used as a control. WSPM2.5 at a concentration of 4μg/mL, 1.2μg/mL, 0.4μg/mL, was added to HBECs for 6 h. One thousandth Blank-DMSO was used as a control. The experiment was divided into seven groups: DMSO control group, TAPM2.5-100 group (57 μg/mL), TAPM2.5-300 group (17.1 μg/mL), TAPM2.5-1000 group (5.7 μg/mL), WSPM2.5-1000 group (4 μg/mL), WSPM2.5-3000 group (1.2 μg/mL), and WSPM2.5-10000 group (0.4 μg/mL). The suffix number of each group name indicates the dilution factor of PM2.5 mother liquor. After culturing for 6 h, the total RNA and protein were collected for the following study.

### Microarray hybridization and data analysis

Total RNA was extracted from HBECs ALI induced with TAPM2.5 or WSPM2.5 at different concentrations according to the design. RNA quantity and quality were measured by NanoDrop ND-1000. RNA integrity was assessed by standard denaturing agarose gel electrophoresis. Arraystar Human LncRNA Microarray V3.0 is designed for the global profiling of human lncRNAs and protein-coding transcripts, which is updated from the previous Microarray V2.0. Approximately 30586 lncRNAs and 26109 coding transcripts can be detected by our third-generation lncRNA microarray. All the microarray analyses were performed by KangChen Bio-tech (Shanghai, China). Sample labeling and array hybridization were performed according to the manufacturer’s instructions. Total RNA was treated with Rnase R (Epicentre, Inc.) to remove linear RNA and amplified and transcribed into uorescent cRNA along the entire length of the transcripts without 3′ bias utilizing a random priming method (Arraystar Flash RNA Labeling Kit, Arraystar) according to the manufacturer’s instructions. Then the labeled cRNAs were purified by RNeasy Mini Kit (Qiagen) according to the protocols provided. The microarray hybridization and the collection of data were performed by KangChen Bio-tech, Shanghai, China. For microarray analysis, Agilent Feature Extraction software (version 11.0.1.1) was used to analyze acquired array images. Quantile normalization and subsequent data processing were performed with using the GeneSpring GX v12.1 software package (Agilent Technologies). After quantile normalization of the raw data, lncRNAs and mRNAs that at least one out of eight samples have flags in Present or Marginal (‘All Targets Value’) were chosen for further data analysis.

### Differential lncRNA and mRNA screen and clustering analysis

Gene Spring software (v. 12.5) was used for normalization of the raw data from each array result. Differentially expressed lncRNA and mRNA were screened with *P*-value less than 0.05 and fold change of more than 2.0. Difference integration analysis (Venn analysis) was then performed to determine the often-characteristic elements. The hierarchical clustering (HCL) was performed to determine the normalized expression level of each RNA type.

### GO and pathway analysis

The differentially expressed mRNAs were submitted to the KEGG database for pathway analysis and were then submitted to the GO database for GO category analysis. The lncRNA function was predicted by GO category analysis and KEGG pathway analysis was performed to analyze the differentially expressed lncRNAs and mRNAs.

### Cell transfection

HBECs were seeded in six wells plates at a concentration of 3 × 10^5^ cells/ml. The siRNA sequences were designed to target the *MEG3* gene, and siRNAs were purchased from GenePharma (GenePharma Co., Ltd., Shanghai, China). The sequences of MEG3 siRNA were 5′-CCC UCU UGC UUG UCU UAC UTT-3′ (sense) and 5′-AGU AAG ACA AGC AAG AGG GTT-3′ (antisense). The sequences of the negative control (NC) siRNA were 5′-UUC UCC GAA CGU GUC ACG UTT-3′ (sense), 5′-ACG UGA CAC GUU CGG AGA ATT-3′ (antisense). Next, 2  ×  10^5^ HBECs cells were seeded in six-well plates and then were transfected with 50 μM MEG3 siRNAs or NC siRNA for 24 h using Lipofectamine™ RNAiMAX (Invitrogen, Carlsbad, CA, U.S.A.). And then the transfected cells were treated with TAPM2.5 and WSPM2.5 for 6 h, respectively.

### Validation of the differentially expressed lncRNAs by qRT-PCR

Total RNA was isolated using TRIzol reagent (Invitrogen) according to the manufacturer’s instructions after indicated treatments. Quantitative real-time PCR (qRT-PCR) assays were performed by using SYBR® Premix Ex Taq™ (Takara, Japan) and PCR were carried out using Applied Biosystems 7500 Real-time PCR System (ABI, U.S.A.). Each individual sample was run in triplicates and the expression level was quantitated using the comparative cycle threshold (*C*_T_) method. The results were normalized to GAPDH expression and RNA enrichments were calculated using the equation 2^−ΔΔ*C*^_T_. The primers of objective genes, GAPDH primers were designed and shown (Table 1).

**Table 1:** Primer sequences used for qRT-PCR analysis

Gene	Sequence (5′–3′)	Product length (bp)
*GAPDH* F	TGTTCGTCATGGGTGTGAAC	154
*GAPDH* R	ATGGCATGGACTGTGGTCAT	
*CCAT1* F	CCTACGCATACCTCTGCTTCT	138
*CCAT1* R	GATTGCTCCTGTTTCCCTTT	
*MEG3* F	CAGGATGGCAAAGGATGAAG	175
*MEG3* R	GCAGGTGAACACAAGCAAAGA	
*HOTAIR* F	AGCCTTTCCCTGCTACTTGT	129
*HOTAIR* R	CTCAAATTCCGGAGCAGCTC	
*GAS5* F	AGCAAGCCTAACTCAAGCCAT	110
*GAS5* R	GTTACCAGGAGCAGAACCATTA	
*MT1JP* F	CCTTGCGGTCTCTCCATTTA	222
*MT1JP* R	CTTCTCCGACGTCCCTTTG	

Abbreviations: CCAT1, colon cancer-associated transcript-1; GAS5, growth arrest-specific transcript 5.

### Western blot assay

Cell lysates were prepared in RIPA buffer (Beyotime, Shanghai, China) containing protease inhibitor cocktail (Roche, Germany) by incubating for 20 min at 4°C. The protein concentrations were determined using a BCA Protein Assay kit (Thermo Fisher Scientific., Rockford, IL, U.S.A.). SDS/PAGE separation gel (10%) was prepared. Equal amount of total proteins was separated by SDS/PAGE (10% gels) transferred on to a PVDF membrane (PerkinElmer, Boston, MA) incubated with primary antibodies overnight. The blots were subsequently incubated with HRP-conjugated secondary antibodies. An Odyssey double color infrared laser imaging system (LI-COR, Lincoln, NE, U.S.A.) was used for scanning and data analyses. GAPDH was used as the internal control for the normalization of the data. The related primary antibodies were anti-GAPDH (dilution 1:2000, Santa Cruz Biotechnology, Santa Cruz, CA), anti-p53 (dilution 1:100, Abcam, Cambridge, MA, U.S.A.; ab28), anti-NF-κB p65 (dilution 1:1000, Abcam, ab76026), anti-mTOR (dilution 1:1000, Abcam, ab2732), anti-β-catenin (dilution 1:500, Abcam, ab6302), anti- caspase-3 (dilution 1:1000, Abcam, ab13586), anti-Bcl-2 (dilution 1:1000, Abcam, ab32124), anti-LC3 (dilution 1:1000, Abcam, ab128025).

### Cell apoptosis assay

The treated cells (1 × 10^6^ cells/ml) were digested, dispersed, centrifuged, collected, washed, re-suspended with 1× binding buffer and double-stained with the annexin-V-FITC/PI staining kit (BD Biosciences). The apoptotic cells were detected by flow cytometry.

### Statistical analysis

Quantile normalization and subsequent data processing were performed using the Kangcheng homemade R software package (Kangcheng Bio-tech, Shanghai, China). Other data were analyzed by the Student’s *t*test and variance (ANOVA) using SPSS 15.0 software (SPSS, Chicago, IL, U.S.A.). Each experiment was repeated at least three times. The data was expressed as the mean ± S.D. *P*<0.05. was considered statistically significant.

## Results

### Expression profiles of lncRNAs and mRNA after WSPM2.5 stimulation

Total RNA was extracted from HBECs exposure for 6 h. Microarray analysis was adopted for the profiling of lncRNAs and mRNAs. In total, 35923 lncRNAs and 24881 mRNAs were detected. Six comparison groups were set according to the differentiation PM2.5 concentration stimulation. DMSO compared with TAPM2.5-100, DMSO compared with TAPM2.5-300, DMSO compared with TAPM2.5-1000, DMSO compared with WSPM2.5-1000, DMSO compared with WSPM2.5-3000, and DMSO compared with WSPM2.5-10000.

**Figure 1:**
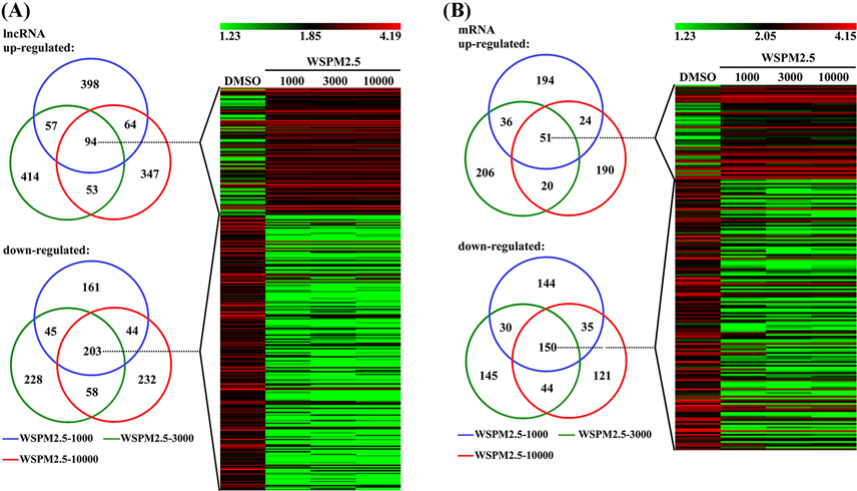
Cluster heat map of all lncRNAs and mRNAs expression for WSPM2.5 stimulation. Cluster heat map of all lncRNAs (**A**) and mRNAs (**B**) expression at different WSPM2.5 concentrations stimulation from microarray data. Often differentially expressed lncRNAs and mRNAs between HBECs at different WSPM2.5 concentrations stimulation. HCL showing often up- and down-regulated lncRNAs and mRNAs amongst the four groups.

For DMSO compared with WSPM2.5-1000, 613 lncRNAs were up-regulated and 453 lncRNAs down-regulated. For DMSO compared with WSPM2.5-3000, 618 lncRNAs were up-regulated and 534 lncRNAs were down-regulated. For DMSO compared with WSPM2.5-10000, 558 lncRNAs were up-regulated and 537 lncRNAs were down-regulated. VENN analysis demonstrated that 94 lncRNAs were up-regulated, and 203 lncRNAs were down-regulated at all WSPM2.5 concentrations. A cluster was generated and analyzed with HCL for the often differentially regulated 94 lncRNAs (up), and 203 lncRNAs (down) at all WSPM2.5 concentration (Figure 1A). As shown in Figure 1B, 305 mRNAs were up-regulated and 359 mRNAs were down-regulated for DMSO compared with WSPM2.5-1000, 313 mRNAs were up-regulated and 369 mRNAs were down-regulated for DMSO compared with WSPM2.5-3000, and 285 mRNAs were up-regulated and 350 mRNAs were down-regulated for DMSO compared with WSPM2.5-10000. VENN analysis demonstrated that 51 mRNAs were up-regulated, and 150 mRNAs were down-regulated at all WSPM2.5 concentration. A cluster was generated and analyzed with HCL for the often differentially regulated 51 mRNAs (up), and 150 mRNAs (down) at all WSPM2.5 concentrations (Figure 1B).

### Expression profiles of lncRNAs and mRNA after TAPM2.5 stimulation

**Figure 2:**
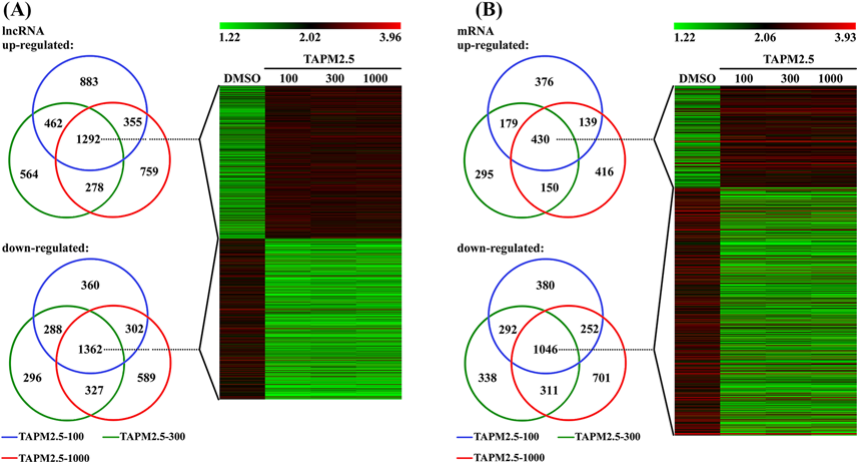
Cluster heat map of all lncRNAs and mRNAs for TAPM2.5 stimulation. Cluster heat map of all lncRNAs (**A**) and mRNAs (**B**) at different TAPM2.5 concentrations stimulation from microarray data. Often differentially expressed lncRNAs and mRNAs between HBECs at different TAPM2.5 concentrations stimulation. HCL showing often up- and down-regulated lncRNAs and mRNAs amongst the four groups.

For DMSO compared with TAPM2.5-100 group, 2992 lncRNAs were up-regulated and 2312 lncRNAs down-regulated. For DMSO compared with TAPM2.5-300, 2596 lncRNAs were up-regulated and 2273 lncRNAs were down-regulated. For DMSO compared with TAPM2.5-1000, 4609 lncRNAs were up-regulated and 2580 lncRNAs were down-regulated. VENN analysis demonstrated that 1292 lncRNAs were up-regulated, and 1362 lncRNAs were down-regulated at all TAPM2.5 concentrations. A cluster was generated and analyzed with HCL for the often differentially regulated 1292 lncRNAs (up) and 1362 lncRNAs (down) at all TAPM2.5 concentration s (Figure 2A). As shown in Figure 2B, 1054 mRNAs were up-regulated and 1987 mRNAs were down-regulated for DMSO compared with TAPM2.5-300, 1135 mRNAs were up-regulated and 2310 mRNAs were down-regulated for DMSO compared with TAPM2.5-1000, and 305 mRNAs were up-regulated and 359 mRNAs were down-regulated for DMSO compared with WSPM2.5-1000. VENN analysis revealed that 430 mRNAs were up-regulated, and 1046 mRNAs were down-regulated at all TAPM2.5 concentrations. A cluster was generated and analyzed with HCL for the often differentially regulated 430 mRNAs (up) and 1046 mRNAs (down) at all TAPM2.5 concentrations (Figure 2B).

### Differentially expressed lncRNAs

**Figure 3:**
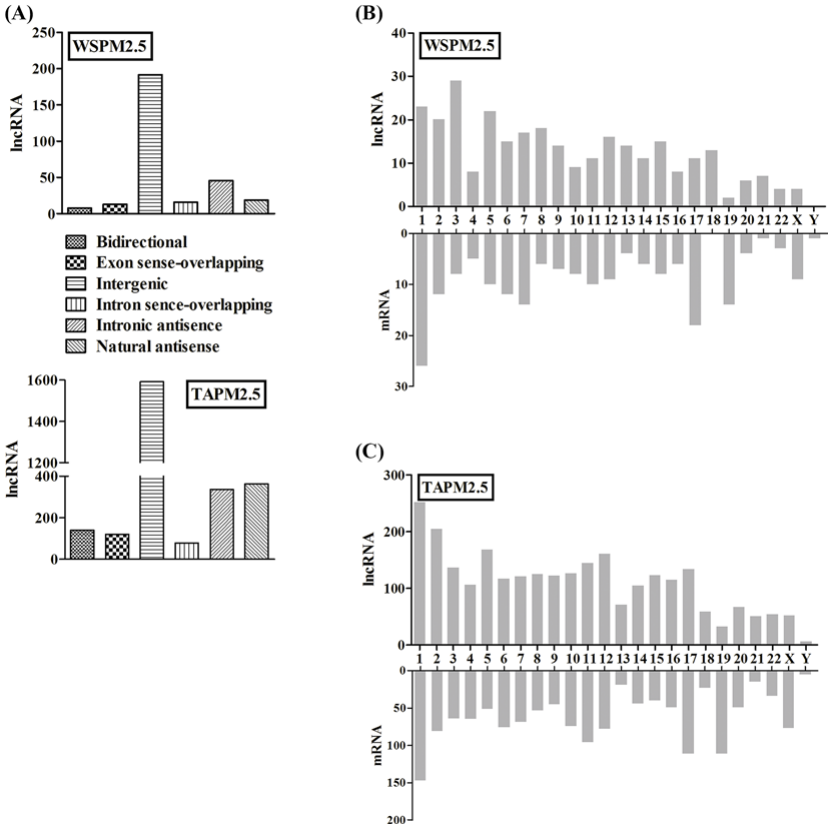
Differentially expressed lncRNAs and chromosomal distribution (**A**) The graph represents the total number of lncRNAs differentially expressed in the array, based on the location and direction. The lncRNAs were subcategorized into bidirectional, exon sense-overlapping, intergenic, intron sense-overlapping, intronic antisense, and natural antisense in both TAPM2.5 and WSPM2.5 treated HBECs**.** Chromosomal distribution of differentially expressed lncRNAs and mRNAs in WSPM2.5 (**B**) and TAPM2.5 (**C**) treated HBECs.

As shown in Figure 3A, the total number of lncRNAs differentially expressed in the array, based on the location and direction, the lncRNAs were subcategorized into bidirectional, exon sense-overlapping, intergenic, intron sense-overlapping, intronic antisense, and natural antisense. Most of the lncRNAs belonged to the kind of intergenic. The differentially expressed lncRNAs and mRNAs were distributed on each of the chromosomes, including the X and Y chromosomes (Figure 3B,C).

### Bioinformatics analysis

**Figure 4:**
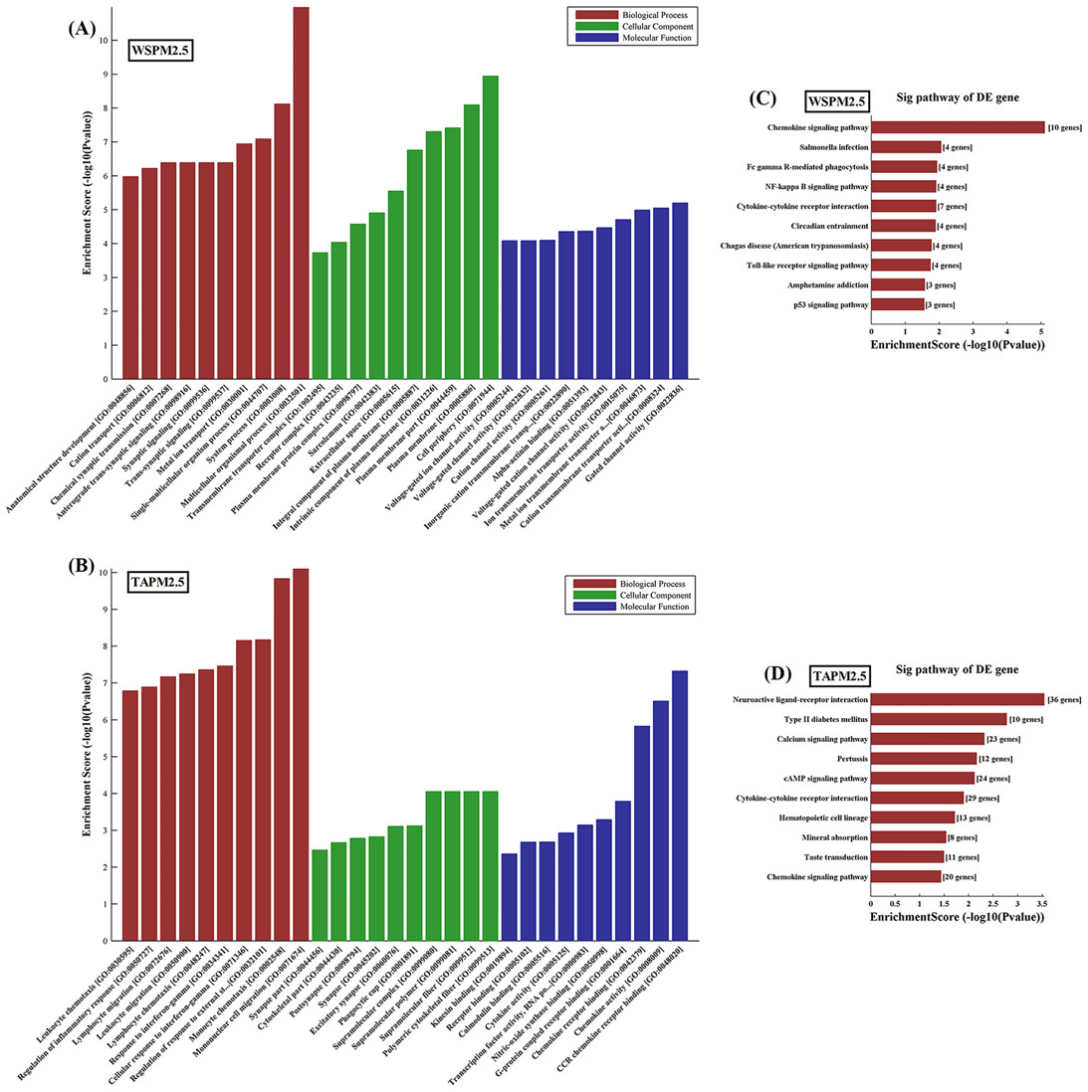
Gene Oncology analysis of lncRNA-target genes according to biological process, cell component, and molecular function Ten most up-regulated GO classifications for WSPM2.5 (**A**) or TAPM2.5 (**B**) treated HBECs compared with control HBECs. (**C**) The top ten pathways that were up-regulated in HBECs induced with WSPM2.5 at different concentrations. (**D**) The top ten pathways that were up-regulated in HBECs induced with TAPM2.5 at different concentrations.

Gene Oncology analysis of lncRNA-target genes was performed according to biological process, cell component, and molecular function. The *P*-value denotes the significance of GO Term enrichment in the differentially expressed mRNA list. The lower the *P*-value, the more significant the GO Term (the *P*-value cut-off is 0.05). The results showed that ten most up-regulated genes respectively involved in biological processes, cellular components, and molecular functions in the WSPM2.5 (Figure 4A) or TAPM2.5 (Figure 4B) treated HBECs.

Pathway analysis based on the latest KEGG database allows users to determine the biological pathways with significant enrichment of differentially expressed mRNAs. The *P-*value denotes the significance of the pathway, with lower *P*-values indicating greater significance (the *P*-value cut-off to determine significance is 0.05). The top ten up-regulated pathways included chemokine signaling pathway, *Salmonella* infection and Fc γ R-mediated phagocytosis in WSPM2.5 treated HBECs (Figure 4C). The top ten up-regulated pathways included Neuractive ligand–receptor interaction, type II diabetes mellitus and calcium signaling pathway in TAPM2.5 treated HBECs (Figure 4D).

### mRNA expression of lncRNAs and protein levels of the target genes

**Figure 5:**
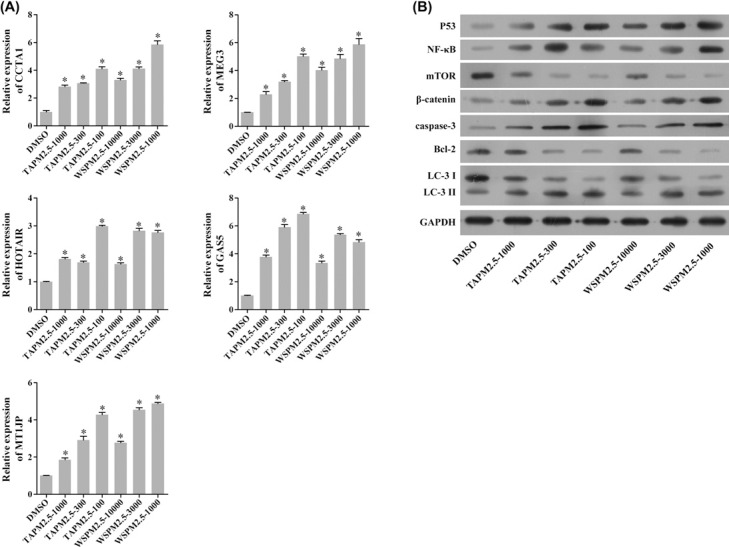
Differential expression of several lncRNAs and the target genes (**A**) The relative mRNA expressions of CCAT1, MEG3, HOTAIR, GAS5, and MT1JP were detected by RT-PCR. The data are expressed as mean ± S.D.; each experiment was replicated three times. Compared with the control, *P<0.05. (**B**) Changes in protein levels in HBE cells treated by the different concentrations of PM2.5 for 6 h. GAPDH was used as the internal control. The data are expressed as mean ± S.D.; each experiment was replicated three times. Compared with the control, *P<0.05.

In order to analyze the concentration-dependent relationship between PM2.5 and the alterations of lncRNAs expression, we detected the expression of lncRNAs in HBECs that were treated with PM2.5 at the same time, but with different treatment concentrations (Figure 5A). Compared with the control group, the expressions of colon cancer-associated transcript-1 (CCAT1), MEG3, HOTAIR, growth arrest-specific transcript 5 (GAS5), and MT1JP were significantly up-regulated in both TAPM2.5 and the WSPM2.5 treatment groups (*P<0.05). An extensive literature indicated that MEG3 plays important roles in lung cancer, such as proliferation, apoptosis and epithelial–mesenchymal transition (EMT) [33–38]. Therefore, we further explored whether lncRNA MEG3 regulates the cell apoptosis and autophagy by regulating cell apoptosis and autophagy associated genes, the expressions of seven genes associated with cell apoptosis and autophagy were detected after 6 h treatment with PM2.5 at different concentrations using Western blot. The results showed that the expression of p53, NF-κB, β-catenin protein, and the apoptosis-associated proteins caspase-3 were significantly increased; however, the autophagy-associated markers and Bcl-2 and LC3 were significantly decreased (Figure 5B). The expressions of the proteins were associated with the concentration of PM2.5 in a dose-dependent manner.

### TAPM2.5 and WSPM2.5 promote apoptosis of HBE cells

**Figure 6:**
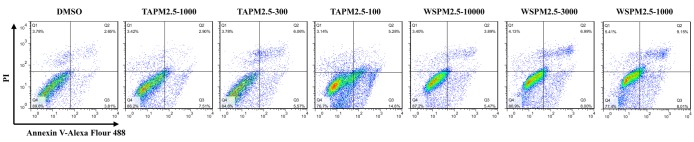
Effect of TAPM2.5 and WSPM2.5 on apoptosis in HBE cells HBE cells were treated by different concentrations of TAPM2.5 and WSPM2.5 for 6 h and the cells were divided into seven groups: DMSO control group, TAPM2.5-1000 group, TAPM2.5-300 group, TAPM2.5-100 group, WSPM2.5-10000 group, WSPM2.5-3000 group, and WSPM2.5-1000 group. Apoptotic cells were detected by flow cytometry.

We further investigated whether TAPM2.5 and WSPM2.5 play critical roles in the apoptosis of HBE cells using flow cytometry. HBE cells were treated with different concentrations of TAPM2.5 and WSPM2.5 for 6 h. The results indicated that with the increase in TAPM2.5 and WSPM2.5 concentrations, the apoptosis abilities were gradually enhanced (Figure 6). Therefore, we showed that TAPM2.5 and WSPM2.5 promoted apoptosis of HBE cells.

### Silencing of MEG3 inhibits apoptosis and autophagy of HBE cells

**Figure 7:**
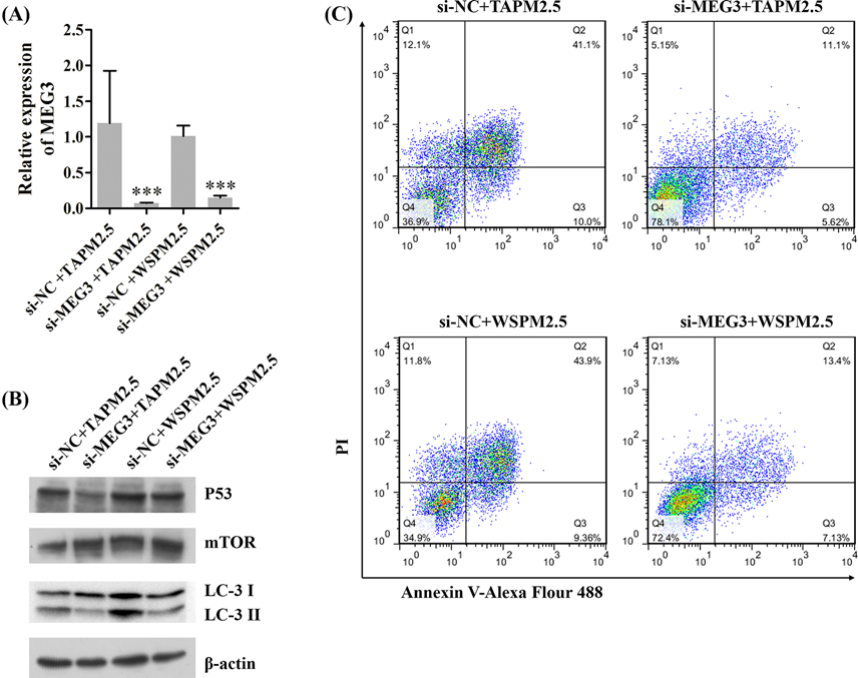
Silencing of MEG3 inhibits apoptosis and autophagy of HBE cells HBE cells were transfected with NC (si-NC) and MEG3 siRNAs (si-MEG3) and then treated with TAPM2.5 and WSPM2.5, respectively. (**A**) The relative expression level of MEG3 was detected by qRT-PCR assay, ***P<0.001. (**B**) Western blot assay was performed to detect p53, mTOR, and LC-3 expressions, β-actin was used as the internal reference. (**C**) Cell apoptosis was examined using flow cytometry.

Further, we explored the roles of MEG3 on the apoptosis and autophagy of HBE cells. HBE cells were transfected with NC (si-NC) and MEG3 siRNAs (si-MEG3), and then treated with TAPM2.5 and WSPM2.5, respectively. Our results showed that silencing of MEG3 significantly decreased the relative expression level of MEG3 mediated by TAPM2.5 and WSPM2.5 (***P<0.001, Figure 7A). We also showed that silencing of MEG3 down-regulated p53 and LC-3 expressions, and up-regulated mTOR expression (Figure 7B). In addition, we demonstrated that the apoptosis rates were decreased in si-MEG3 group compared with si-NC group (Figure 7C). Therefore, we suggested that silencing of MEG3 inhibited apoptosis and autophagy of HBE cells.

## Discussion

PM2.5 is an important component of air pollution. It is composed of large amounts of heavy metals, organics, inorganics, microorganisms, and other minor substances. Studies have shown that substances such as benzoapyrene (BaP) and dioxin are carcinogenic and mutagenic [39,40]. Epidemiological studies have confirmed that prolonged exposure to PM2.5 induced a number of pathological respiratory diseases, such as bronchitis, asthma, and COPD. Statistics showed that the atmospheric PM2.5 concentration increases by 10 mg/m^3^, the mortality of respiratory diseases is increased by 2% and the hospitalization rate in patients with COPD is increased by 1.72–6.87% [41,42].

Gene microarray analysis is a useful approach to detect the pathogenic mechanisms of PM2.5 and the relation between molecular mechanisms and biomarkers. Xu et al. [22] and Liu et al. [43] have reported that lncRNAs play important functions in the regulation of biological processes such as the cell cycle, proliferation, and apoptosis. In the present study, we assessed the effects of PM2.5 on lncRNA and target gene expression. We exposed HBECs to two types of PM2.5 arterial TAPM2.5 and WSPM2.5 and performed lncRNA microarray analysis. According to the results of our microarray analysis, we selected differentially expressed lncRNAs induced by TAPM2.5 and WSPM2.5 exposure.

To further characterize the role of PM2.5 on lncRNA expression, according to the results of lncRNAs microarray, five up-regulated lncRNAs, CCAT1, MEG3, HOTAIR, GAS5, and MT1JP, were selected for further studies. HOTAIR is known to repress the HOXD locus gene transcription in *trans*, regulate chromatin states and epigenetic inheritance [44,45]; MT1JP a novel tumor suppressor, which appears to play a key regulatory role upstream of p53 [46]. CCAT1, which was first found to be up-regulated in colon cancer [18], and de-regulated in various cancer types, including lung cancer [47–51]. The lncRNA GAS5 was significantly down-regulated in NSCLC tissues and cell lines [52]; MEG3, which was identified to be up-regulated and played as a tumor suppressor gene in a lot of tumors [53–56]. To confirm the reliability and validity of the microarray results, we used qRT-PCR to validate the expression patterns of the five lncRNAs and the qRT-PCR results matched well with the microarray data. We also observed that differentially expressed lncRNAs and mRNAs were distributed on each of the chromosomes including the X and Y chromosomes and display different quantities in PM2.5-induced cell damage. In addition, most of the lncRNAs belonged to the kind of intergenic.

GO and KEGG analyses were performed to further annotate the biological functions of differentially expressed lncRNAs and their target genes. lncRNA MEG3 was chosen for further functional studies as well. lncRNAs are widely expressed and known to have a mechanism of action upon activation of common transcription factors such as NF-κB, Sox2, p53, Oct4, b-catenin, mTOR, and Nanog [57]. Deng et al. [58] and Xiong et al. [60] showed that apoptosis was a critical endpoint to assess toxicity of particulate matter and mitochondria-mediated apoptosis was an important apoptotic pathway initiated by caspase-3 activating [59]. Autophagy is a predominant cellular process and recent research has revealed that autophagy is involved in PM2.5 induced-cardiovascular and respiratory diseases [58,61]. Recently, a study reported that the process of apoptosis and autophagy play potential role in PM2.5-induced ocular corneal diseases and cytotoxicity of PM2.5 in HBECs [59].

Based on these findings, we hypothesized that apoptosis and autophagy may also be critical in PM-induced COPD and indicated that the function of lncRNA MEG3 in cell apoptosis and autophagy regulation might be mediated by certain cell apoptosis and autophagy-associated proteins. We therefore determined the expressions of seven common cell-apoptosis and autophagy proteins by Western blot analyses. With increased PM2.5 concentration, the protein level of p53, apoptosis-associated proteins caspase-3 significantly increased; however, the expression of autophagy proteins Bcl-2 and LC3 was significantly decreased. Therefore, we suggested that PM2.5 exposure induced HBECs apoptosis and autophagy and the PM2.5-induced cell damage of HBECs occurred in a dose-dependent manner.

NF-κB, which serves as one of nuclear transcription factors, is found in eukaryotic cells and regulates DNA transcription [62]. Studies have also indicated that NF-κB plays critical roles in biological processes including proliferation, differentiation, migration, and metastasis [63,64]. Wnt/β-catenin signaling pathway is involved in the disruption of β-catenin destruction complex by phosphorylation of glycogen synthase kinase-3β (GSK-3β), and causes cytosolic accumulation, nuclear translocation of β-catenin and tumorigenesis [65,66]. Activation of NF-κB and Wnt/β-catenin signaling pathways has been demonstrated to participate in multifarious malignant tumors [67,68]. The serine/threonine kinase mTOR acts as a key component of the mTORC1 complex, which exerts significant effects in various diseases [69–71]. It has been indicated that silencing of MEG3 promotes cisplatin resistance of lung cancer via Wnt/β-catenin signaling pathway [38]. P53 acts as a tumor suppressor and participates in many biological processes including cell proliferation, cell cycle arrest, apoptosis, migration, metastasis, autophagy, and DNA repair processes [72–74]. Previous studies have demonstrated that p53 can be activated by lncRNA MEG3 [75]; lncRNA MEG3 is involved in proliferation and metastasis of gastric cancer by p53 [76]; lncRNA MEG3 inhibited NSCLC cells proliferation and promoted apoptosis by p53 [36]; lncRNA MEG3 can be interacted with p53 in hepatoma cells [77]. In our study, we demonstrated that MEG3 up-regulated p53, NF-κB, β-catenin, and caspase-3 expressions, and down-regulated Bcl-2 and LC3 expressions. In addition, we showed that silencing of MEG3 inhibited apoptosis and autophagy of HBE cells.

In conclusion, we demonstrated that lncRNA MEG3 was up-regulated in HBECs and mediated cell apoptosis and autophagy by increasing the expression of p53 in HBECs treated with PM2.5. Therefore, lncRNA MEG3 could be a promising target for COPD therapy.

## Abbreviations

AECII: type II alveolar epithelial cells
ALI: air–liquid interface
FEV1: forced expiratory volume in the first second
FVC: forced vital capacity
CCAT1: colon cancer-associated transcript-1
COPD: chronic obstructive pulmonary disease
*C*_T_: cycle threshold
EMT: epithelial–mesenchymal transition
GAS5: growth arrest-specific transcript 5
GO: gene ontology
HBE: human bronchial epithelial
HBEC: human bronchial epithelial cell
HCL: hierarchical clustering
KEGG: Kyoto Encyclopedia of Genes and Genomes
lncRNA: long non-coding RNA
MEG3: maternally expressed gene 3
NC: negative control
NSCLC: nonsmall cell lung cancer
PM2.5: fine particulate matter
qRT-PCR: quantitative real-time PCR
SAL: senescence-associated lncRNAs
TAPM2.5: traffic ambient PM2.5
WSPM2.5: wood smoke PM2.5
